# Ultrasound-Guided Fascial Hydrorelease for Persistent Pain After Hamstring Injury

**DOI:** 10.3390/jfmk10030318

**Published:** 2025-08-15

**Authors:** Kousuke Shiwaku, Hidenori Otsubo, Daiki Nishikawa, Rikiya Itagaki, Hiroyuki Takashima, Gakuto Nakao, Tomoaki Kamiya, Daisuke Suzuki, Makoto Emori, Carla Stecco, Atsushi Teramoto

**Affiliations:** 1 Department of Orthopaedic Surgery, Sapporo Medical University, Sapporo 0608543, Japan; shiwaku1008@gmail.com (K.S.); dnishikawa0110@gmail.com (D.N.); rikiya.i.0829@gmail.com (R.I.); tkamiya0606@gmail.com (T.K.); memori@sapmed.ac.jp (M.E.); teramoto.atsushi@gmail.com (A.T.); 2 Department of Orthopaedic Surgery, KKR Sapporo Medical Center, Sapporo 0620931, Japan; 3 Department of Orthopaedic Surgery, Sapporo Sports Clinic, Sapporo 0600041, Japan; 4 Department of Orthopaedic Surgery, Obihiro Kyokai Hospital, Obihiro 0800805, Japan; 5 Division of Biomedical Science and Engineering, Hokkaido University, Sapporo 0600808, Japan; hirota-kashima@pop.med.hokudai.ac.jp; 6 Graduate School of Health Sciences, Sapporo Medical University, Sapporo 0608543, Japan; gakunn.24@gmail.com; 7 Chitose Rehabilitation College, Chitose 0660055, Japan; daisuke@sapmed.ac.jp; 8 Department of DNS, Padova University, 35122 Padova, Italy; carla.stecco@unipd.it

**Keywords:** hydrorelease, perineural fascial pain, hamstring injury, fascial thickening, posterior femoral cutaneous nerve, ultrasound-guided injection

## Abstract

**Background:** Post-hamstring-injury residual pain may persist despite muscle-tissue healing and impairs athletes seeking early full recovery. Given their unclear cause, recent attention has focused on the role of fascial dysfunction and a method to restore fascial mobility, namely, hydrorelease (HR), involving the ultrasound (US)-guided injection of saline. We evaluated the clinical efficacy of HR for treating residual pain and ascertained the underlying pathological mechanisms. **Methods**: Seven patients (aged 17–49 years) with residual pain ≥8 weeks after hamstring injury were included. All exhibited localized tenderness and US findings of fascial thickening around the aponeurotic fascia (APF). HR with 6.0 mL saline–lidocaine solution (0.17% lidocaine) was performed and targeted the peri-APF loose connective tissues. Pain was evaluated using a numerical rating scale (NRS) before and after HR. Passive straight leg raise (SLR) was used to assess tightness. **Results**: Post-HR, the mean NRS score significantly decreased from 10 to 0.86 (*p* = 0.017). Four patients required a single HR session; three required two–four sessions. Post-HR, the tightness of all patients improved. Short-axis US of the posterior thigh revealed APF fascial thickening in the area of tenderness, including the posterior femoral cutaneous nerve (PFCN). No adverse events or recurrence occurred during the follow-up (mean: 6.6 months). **Conclusions**: HR targeting the peri-PFCN-APF effectively reduced residual pain following hamstring injury. These findings support the concept of “Perineural fascial pain”—a pathology wherein persistent pain originates not from direct nerve damage or classical myofascial pain syndrome but rather from the dysfunction of the surrounding fascia.

## 1. Introduction

Hamstring injuries are common sports injuries that remain a challenging clinical problem, particularly for athletes aiming for early return to play [1]. Despite initial recovery, approximately one-third of patients experience recurrence [2] with an average recovery period of 6–8 weeks before returning to play [3]. In most cases, athletes return to sports without significant residual symptoms. However, in some patients, pain and tightness that persist for several months after injury interfere with return to play. Such cases are rarely documented in the literature. In a previously reported case, fascial thickening between the epimysial layers was observed at the site of tenderness, and the patient’s symptoms improved following the hydrodissection (HD) [4]. HD involves injecting a low-invasive mixture of anesthetics (e.g., bupivacaine, lidocaine), corticosteroids, and saline to mechanically separate fascial layers and peripheral nerves from surrounding structures [5,6,7]. HD is designed to mechanically “separate” peripheral nerves from surrounding structures and generally requires larger fluid volume (10–50 mL). In recent years, a less-invasive technique called hydrorelease (HR) has gained attention. HR involves ultrasound(US)-guided injection of saline or diluted local anesthetic into the fascial layers, without using corticosteroids or pharmacologic anesthesia. The aim is to restore fascial mobility and reduce mechanical restrictions [8,9,10,11]. HR is performed by injecting fluid into the fascial planes, including the aponeurotic fascia (APF), interfascial loose connective tissue, or intramuscular region [8,9,11]. Importantly, HR is conceptually and technically distinct from HD. While HR aims to “loosen” or “peel” fascial layers using small fluid volumes (2–10 mL), HD is designed to mechanically separate structures using larger volumes. A biomechanical study [10] showed that HR can significantly reduce the gliding resistance between fascial layers, suggesting a potential mechanism for its clinical effects. Recent advances in ultrasound imaging enabled visualization of fascial thickening, stacking, and densification [12,13]. Consequently, HR has been applied to cases of peripheral nerve disorders and myofascial pain syndrome (MPS), targeting tender points and hyperirritable fascial zones (trigger points) [8,14,15].

Based on these considerations, we hypothesized that HR could be effective in treating residual pain after hamstring injuries by restoring fascial mobility and reducing restrictions. The aim of this study was to investigate the clinical efficacy of HR for treating residual pain after hamstring injuries and to explore the potential underlying pathological mechanisms.

## 2. Materials and Methods

### 2.1. Patients

Patients experiencing residual myofascial pain in the posterior thigh around the injured site for 8 weeks or more following hamstring injury were recruited for this case series between August 2023 and February 2025. Patients with US-confirmed thickening, stacking, and densification of the APF observed at the site of tenderness of the residual pain were considered suitable for HR. These patients were assessed using a pain scale and underwent flexibility or tightness assessment and ultrasonography. Patients with neuromuscular disease or acute lower extremity musculoskeletal injuries were excluded. All patients received comprehensive information about the purpose and procedures of the study and signed an informed consent statement prior to their participation in the study. This study was approved by our institution (approval number: 2020-26) and adhered to the requirements of the Declaration of Helsinki.

During the study period, seven patients (three women and four men; age, 26.0 (range: 17–49) years; height, 168.0 (range: 159–175) cm; and body weight, 62.1 (range: 48–70) kg) with hamstring injuries and pain persisting for more than 8 weeks visited our hospital, and all of them were ultimately included in the study. All participants were first-time cases with no prior history of hamstring pain or injury. The sports disciplines varied widely and included badminton, marathon running, baseball, hurdling, soccer, short-distance sprinting, and physical training for police officers (running and weight training). The average training experience was approximately 11.6 years (range: 3–27 years), with five participants engaged in competitive club activities and two at the recreational level. Before treatment, all participants reported pain during sprinting or running; one case was unable to return to play, while the remaining six continued to participate despite persistent pain. Following treatment, all participants experienced complete resolution of pain and fully returned to their sporting activities, including sprinting and competition.

### 2.2. Assessment of Pain During Activity

In the first visit, pain experienced during activity was quantified using a numerical rating scale (NRS) ranging from 0 to 10, with 0 representing no pain and 10 indicating the most severe pain similar to the first visit. For consistency in evaluation, all patients were instructed to rate their pain using a numerical rating scale (NRS) from 0 to 10, where 10 corresponded to the pain intensity experienced at the initial visit. Pain was assessed during the activity that elicited the greatest pain among three standardized tasks. First, hamstring stretching was performed in a standard clinical passive stretch manner in the supine position, with the hip flexed and the knee gradually extended to the point of first pain/first resistance, then held for 5 s. Second, isometric knee flexion was performed with the knee at 30° flexion on the injured side; participants were instructed to perform a maximal voluntary isometric contraction (“as hard as you can”) for 5 s with standardized verbal encouragement; no dynamometry was used, and intensity was standardized by instruction and hold time. Third, light jogging was performed at a self-selected easy pace on a flat corridor under 10 m. This approach enabled standardized comparison of pre- and post-intervention scores. For each patient, all evaluations were performed by the same examiner to ensure consistency. Pain levels were recorded at two time points: before the injection and 5 min after the final HR.

### 2.3. Assessment of Tightness

Tightness was evaluated using the passive straight leg raise (SLR) for both legs. For the SLR angle evaluation, the hip joint of the patient was passively flexed in the supine position, and the hip angle was measured using a goniometer with a line parallel to the trunk as the basic axis and the femur as the moving axis. The maximum flexibility intensity was defined immediately before the subjective pain threshold and without compensatory movements.

Patients without any difference were considered as having no tightness. Patients without any difference in the SLR angle but with discomfort or a feeling of tension were evaluated as having slight tightness. Patients with a difference in the SLR angle in the injured leg compared with the contralateral leg were considered to have significant tightness. Furthermore, the SLR angle was evaluated in patients who had significant tightness. These assessments of tightness were performed both before and 5 min after the HR.

### 2.4. US Imaging and Sonopalpation

**Figure 1:**
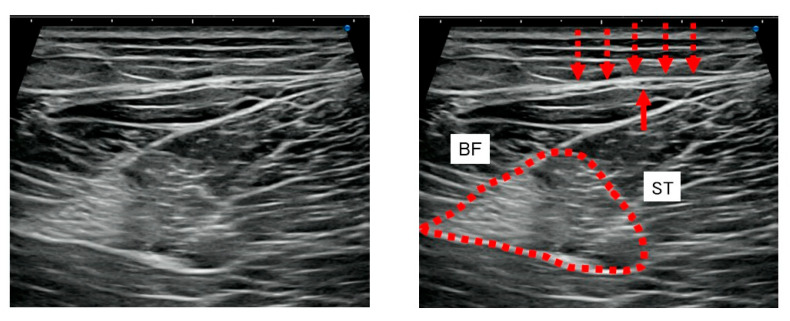
Short-axis ultrasound of the posterior thigh. Fascial thickening (dotted arrows) is observed in the aponeurotic fascia (APF), including the region traversed by the posterior femoral cutaneous nerve (arrow). A slight trace of hematoma is noted in the deeper layer of the hamstring muscle (dotted surrounded area). APF: aponeurotic fascia; BFlh: biceps femoris long head; BFsh: biceps femoris short head; EPI: epimysium; LCT: loose connective tissue; PFCN: posterior femoral cutaneous nerve; SF: superficial fascia; ST: semitendinosus; SM: semimembranosus; RF: rectus femoris; VI: vastus intermedius; VL: vastus lateralis; VM: vastus medialis.

US (SNiBLE; Konica Minolta, Tokyo, Japan) imaging was thoroughly performed, both in short-axis and long-axis, widely around the sites of tenderness in the posterior aspect of the thigh of the injured leg and a similar area of the contralateral leg. Although it cannot be quantitatively evaluated, sonopalpation was performed to assess the depth of tenderness. In patients reporting tenderness on palpitation of the layer surrounding the APF, this layer was identified as the target of HR (Figure 1).

### 2.5. US-Guided Hydrorelease

**Figure 2:**
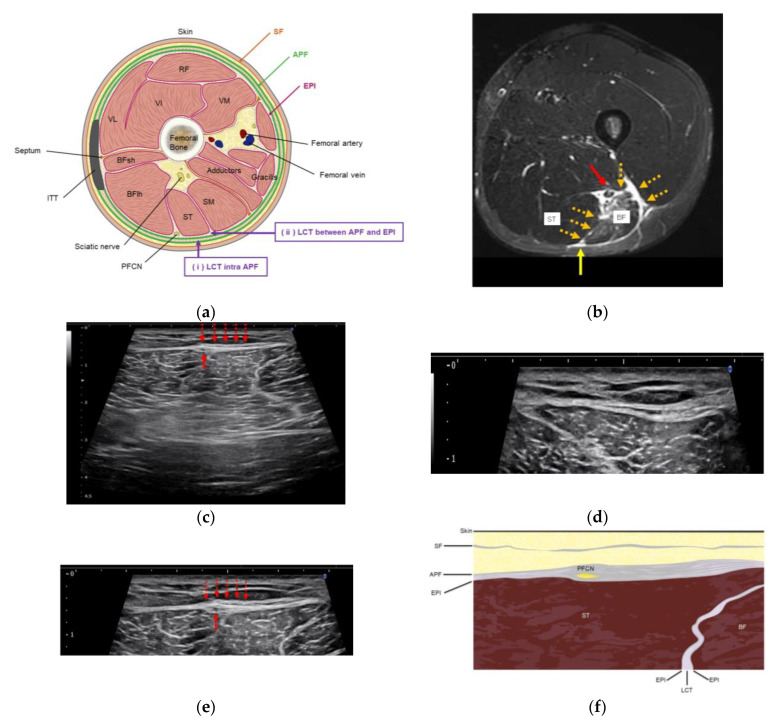
(**a**) Illustration of the posterior thigh anatomy. The posterior femoral cutaneous nerve is located within the LCT between the APF and the EPI of the ST and BF. (**b**) Illustrative image from a non-study participant. Axial STIR MRI of the right thigh 3 days after injury. Bleeding is observed extending through the loose connective tissue, potentially contributing to subsequent fascial thickening of the APF. The red arrow indicates the suspected site of the original hamstring injury; the orange dotted arrow indicates the loose connective layer through which the bleeding propagated; and the yellow arrow indicates the posterior femoral cutaneous nerve. Repetition time/echo time, 6750 ms/60 ms; Inversion time, 150; matrix, 256 × 224; echo train length, 12; slice thickness/gap, 6 mm/2 mm. (**c**) Ultrasound image (affected side, pretreatment): The posterior femoral cutaneous nerve (red arrow) is located beneath the APF and above the epimysium of the ST, prior to the hydrorelease. (**d**) Ultrasound image (contralateral side, zoomed): Normal fascial anatomy of the contralateral thigh is shown as a reference for comparing fascial layers and nerve position. (**e**) Ultrasound image (affected side, pretreatment, inset): Thickened APF and underlying epimysium are visualized with clearer nerve-fascial relationships. (**f**) Concept illustration of perineural fascial pain: The schematic highlights the relationships between the posterior femoral cutaneous nerve and surrounded fascia (APF and the epimysium of the ST). APF: aponeurotic fascia; BF: biceps femoris; EPI: epimysium; ITT: iliotibial tract; LCT: loose connective tissue; MRI: magnetic resonance imaging; PFCN: posterior femoral cutaneous nerve; SF: superficial fascia; SM: semimembranosus; ST: semitendinosus; STIR: slice short τ inversion recovery; ST: semitendinosus.

**Figure 3:**
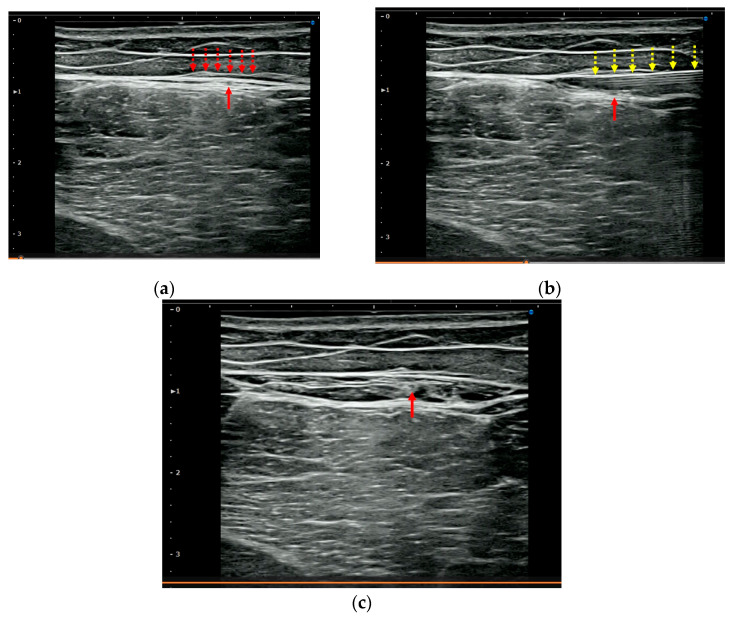
Short-axis ultrasound of the posterior thigh before (**a**), during (**b**), and after (**c**) HR. (**a**) Fascial thickening (dotted arrows) in the APF, including the posterior femoral cutaneous nerve (arrow), is evident prior to treatment. (**b**) A 25 G, 40 mm needle (yellow dotted arrows) is visible in the superficial layer of the APF, with the posterior femoral cutaneous nerve (red arrow) just below. Saline was injected at a superficial level relative to the nerve. (**c**) The posterior femoral cutaneous nerve (red arrow) is located within the fascial layers, surrounded by saline injected during HR. APF: aponeurotic fascia; HR: hydrorelease.

HR was performed using a short-axis view and an in-plane technique. The procedure involved injecting 5.0 mL saline mixed with 1.0 mL 1.0% lidocaine (total: 6.0 mL (0.17%) lidocaine), without a significant anesthetic effect, using a 25 G 40 mm needle. The needle was directed toward the site with hyperechoic changes surrounding the APF. The target points were the loose connective tissues within the APF and between the aponeurotic and epimysial fasciae (Figure 2a). Figure 3 shows an example of before- and after-HR scans. HR was performed at the site of tenderness and was eventually performed between the proximal and distal thirds of the thigh for all patients. HR was performed by two orthopedic surgeons certified by the Japanese Orthopedic Association, each with more than 8 years of experience in performing musculoskeletal US.

One week after HR, patients were asked to evaluate pain at the HRt site using the NRS. HR was repeated only in patients with an NRS pain score ≥ 3 and was performed for an additional one-to-four sessions until the NRS pain score decreased to ≤3. In addition to HR, all patients received conservative treatment, such as rehabilitation.

### 2.6. Statistical Analysis

Changes in pain levels before and after the final HR were analyzed. All statistical analyses were performed using SPSS Statistics software (v.28.0, IBM Corp., Armonk, NY, USA). The Wilcoxon signed-rank test was used to compare NRS scores before and after HR. Statistical significance was set at 5% for all tests.

## 3. Results

### 3.1. Assessment of Pain During Activity

**Figure 4:**
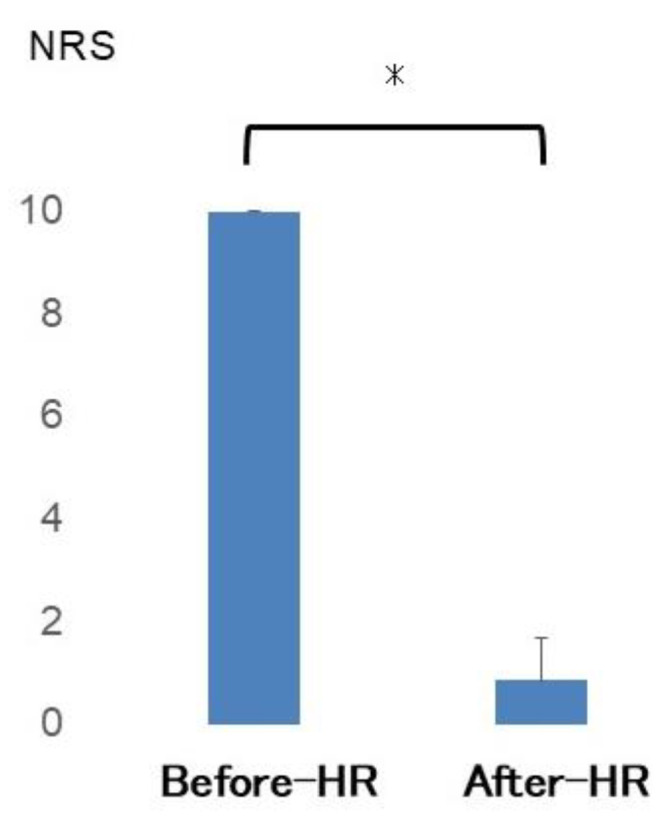
NRS before and after HR, with 0 representing no pain and 10 indicating the most severe pain similar to the first visit. NRS: numerical rating scale; HR: hydrorelease. *: significantly different.

The mean NRS score (Figure 4) significantly decreased from 10 before the HR to 0.86 after the HR (*p* = 0.017).

Four patients required only a single HR injection, whereas repeated HRs were performed in three patients. Specifically, the second HR was performed for two patients, and a second, third, and fourth HRs were performed for one patient. HR injections were performed at 2.6 sites (range: 1–4 times) on average at each visit. No recurrence was observed after an improvement following the final HR for 6.7 (range: 3–12) months.

### 3.2. Assessment of Tightness

Three patients had slight tightness before HR and no tightness after HR. The remaining four patients reported significant tightness before HR. They exhibited 20° (range: 15–30°) of SLR restriction compared with the contralateral side. After the final HR, the restriction improved by 3.8° (range: 0–5) compared with the contralateral leg.

### 3.3. US Imaging and Sonopalpation

US imaging showed a similar tendency in all cases included in this study. The short-axis US of the posterior thigh revealed greater fascial thickening at the tender site of the APF. The posterior femoral cutaneous nerve (PFCN) was found to traverse this thickened region (Figure 2c–e), and there was a trace of hematoma in the deeper layer of the hamstring muscle (Figure 1).

## 4. Discussion

In this study, all patients with residual pain following a hamstring injury demonstrated localized tenderness. On US, this corresponded to fascial thickening confined to the APF and adjacent epimysium. The PFCN was observed traversing this thickened region. US-guided HR was selectively applied to this fascial layer, resulting in marked symptom improvement in all cases. This therapeutic response suggests that a deep hematoma that was associated with the initial hamstring trauma was not the source of the pain; rather, the pain was attributable to mechanical dysfunction around the nerve, which was caused by surrounding fascial abnormalities and, particularly, impaired fascial gliding.

Regarding the development of fascial thickening around the PFCN, we hypothesize the following mechanism: As illustrated in Figure 2b, bleeding from the deep site of muscle injury may have extended toward the more superficial APF layer via the loose connective tissue. During the acute phase, pain-induced movement limitation and relative immobilization may promote fascial densification, adhesion, and restricted mobility. Over time, such changes can lead to structural thickening of the fascia, likely interfering with the gliding of the PFCN and causing persistent symptoms.

Several mechanisms may explain the clinical efficacy of HR in relieving symptoms.

First, as demonstrated in the biomechanical study [10], HR restores fascial mobility by reducing gliding resistance. This may decrease mechanical tethering and associated stress on the posterior femoral cutaneous nerve. Second, fascial thickening can lead to microvascular compression and localized edema, contributing to heightened pain sensitivity. HR may improve circulation and oxygenation, thereby normalizing metabolic function and raising the pain threshold. Third, unlike nerve blocks, HR acts indirectly by modifying the nerve’s surrounding fascial environment, offering a minimally invasive approach for both structural and functional recovery.

Clinical studies have reported improved shoulder motion and pain after HR targeting thickened fascia in the frozen shoulder, as well as rapid symptom relief in patients with acute low back pain. Collectively, these findings support the concept that restoring fascial mobility is a critical component of pain management, even when no direct neural pathology is present [8,9,10].

The PFCN originates from the S1 to S3 and transverses the deep gluteal space. It then emerges superficially to the skin of the posterior thigh [16]. Distal to the ischial tuberosity, the PFCN exhibits variable branching patterns. Ultimately, the PFCN contributes a perineal branch, the inferior cluneal nerves, and the posterior thigh cutaneous branches [16,17]. The efficacy of HD of the PFCN around the deep fascial layer of the gluteus maximus has been demonstrated [18]. Although the speculated pathological sites differ from those involved in HR in this study, these findings, along with those of our study, highlight the clinical relevance of the PFCN and warrant further investigation into its involvement in pain conditions.

Based on these findings, we propose the term “Perineural Fascial Pain (PFP)” to describe a clinical entity characterized by mechanical pain originating not from direct nerve injury but from pathological changes in the fascia surrounding peripheral nerves. In this condition, the perineural fascia becomes dehydrated, fibrotic, or densified, impairing its gliding ability and resulting in traction or compression on the nerve and the surrounding perineural fascia during movement (e.g., stretching or active contraction). This gliding dysfunction leads to nociceptive pain without structural damage to the nerve itself and is often rapidly reversible with ultrasound-guided HR. In our study, the immediate symptom relief following HR supports the notion that fascial pathology, particularly involving the perineural fascial layers, plays a primary and modifiable role in such pain. Further studies are warranted to investigate objective diagnostic tools, such as elastography and dynamic ultrasound, to evaluate fascial sliding and structural characteristics [14,19].

### Limitations

This study had some limitations that should be acknowledged. First, the number of patients included in this study was relatively small, as we included only seven patients, which may limit the generalizability of the findings. Future studies with larger sample sizes are required to further support our findings and draw more definitive conclusions. Second, this study lacked a control group. Thus, no comparison with a non-intervention group could be conducted, which makes it difficult to attribute the observed changes in pain and mechanical properties solely to HR. Future studies consisting of control groups would provide further evidence supporting the efficacy of HR. Third, patients were not followed from the onset of the first injury as the study design involved patients with chronic residual pain subsequent to hamstring injury. Thus, almost all the patients included in this study had visited other hospitals initially and were then referred to our hospital. Fourth, MRI was not performed at the time of injury; thus, it was impossible to accurately classify the type of muscle strain. Fifth, no acute-phase imaging was available for any of the participants. All patients had initially been treated at local clinics and were referred to our facility after persistent symptoms failed to improve, with a mean delay of 10.4 weeks post-injury. Therefore, baseline MRI data were not obtainable. A prospective registry is currently underway to collect acute MRI and serial ultrasound findings for future correlation with chronic fascial alterations. Sixth, ultrasound evaluations were performed by two experienced operators who were aware of the patients’ clinical background; therefore, full blinding was not feasible and may have introduced assessment bias. Seventh, the duration of action of the injected solution remains uncertain, including how long the fluid remains within the targeted fascial layer. Moreover, it is unclear whether the observed effects are transient or contribute to sustained improvement. These issues regarding the mechanism of action and long-term efficacy warrant further investigation in future studies. Eighth, the follow-up period was relatively short, allowing for assessments to be performed immediately after HR and again after one week, which left the evaluation of the potential long-term effects on the mechanical properties of tissue, pain, and functionality unresolved. Future studies should include a longer follow-up period to better define the long-term outcome and durability of treatment effects. Finally, this study did not control for potential co-interventions during the intervention period. Although participants were instructed to continue their usual activities, some may have received additional rehabilitation targeting neural or fascial components, which could have contributed to the observed clinical improvements. For instance, neurodynamic nerve gliding techniques have been shown to increase hamstring flexibility by reducing neural tension and enhancing strain in the surrounding connective tissue [20,21]. Similarly, various myofascial therapies, including stretching and manual myofascial release, have been reported to decrease fascial stiffness and pain [22,23]. These interventions may exert additive or synergistic effects when combined with HR. However, the extent to which such co-interventions influenced the present results remains unclear. Future studies should control for or systematically investigate the combined effects of HR with neural or fascial rehabilitation techniques to clarify their independent and interactive contributions to clinical outcomes.

Despite its limitations, this study had two major strengths. First, it highlights the need to consider post-muscle-injury residual pain, a condition that is largely overlooked in the literature. Second, this study specifically examined residual pain after hamstring injury, which is considered to be related to a newer concept: “PFP.” Because hamstring injury typically results from a single, well-defined traumatic event, the pathophysiological process is often more isolated and temporally distinct. Residual pain after hamstring injury is easier to analyze and may contribute to a deeper understanding of the pathology of myofascial pain than other chronic conditions such as shoulder stiffness and lower back pain, which tend to recur due to repeated mechanical loading during daily activities and are highly multifactorial. Further research is necessary to investigate the underlying mechanisms of myofascial pain and other pathologies and to determine the appropriate treatment indications. Furthermore, investigating the applicability of HR to other myofascial pain syndromes and chronic pain conditions may increase the generalizability of this treatment approach. In general, HR is considered useful not only as a therapeutic technique but also as a diagnostic approach to elucidate pathology. In this case, valuable information was recorded showing that the layer surrounding the APF plays a significant role in the etiology of residual hamstring pain. The results of this study suggest that the fascial layer around the APF and adjacent epimysium may be involved in pain in similar pathologies. Widespread adoption of such diagnostic approaches is expected to improve our understanding of the unexplained pain pathology and ultimately advance the elucidation of the underlying mechanisms.

## 5. Conclusions

This study demonstrated that in patients with residual “perineural fascial pain” following hamstring injury, fascial thickening of the APF and involvement of the posterior femoral cutaneous nerve were consistently observed. HR that targeted the aponeurotic fascia-associated fascial layer was effective in alleviating symptoms, and this suggests that residual pain may be attributable to altered fascial dynamics rather than nerve or muscle pathology alone. Further large-scale controlled trials with long-term follow-up are needed to validate these findings.

## Data Availability

The original contributions presented in this study are included in the article. Further inquiries can be directed to the corresponding author(s).

## Abbreviations

The following abbreviations are used in this manuscript:

APF	Aponeurotic Fascia
HD	Hydrodissection
HR	Hydrorelease
MPS	Myofascial Pain Syndrome
MRI	Magnetic Resonance Imaging
NRS	Numerical Rating Scale
PFCN	Posterior Femoral Cutaneous Nerve
PFP	Perineural Fascial Pain
SLR	Straight Leg Raise
STIR	Short Tau Inversion Recovery
US	Ultrasound
